# Supplementary material for: Multilayered regulations of alternative splicing, NMD, and protein stability control temporal induction and tissue-specific expression of TRIM46 during axon formation
Source: Nat Commun. 2022 Apr 19;13:2081. doi: 10.1038/s41467-022-29786-4 (PMC9019110; doi:10.1038/s41467-022-29786-4)
Supplement: Supplementary file 1 — Supplementary Information [file 41467_2022_29786_MOESM1_ESM.pdf]

SUPPLEMENTARY INFORMATION

Multilayered Regulations of Alternative Splicing, NMD, and Protein Stability Control Temporal Induction and Tissue-specific Expression of TRIM46 during Axon Formation

John K. Vuong[1], Volkan Ergin[1], Liang Chen[2], Sika Zheng[1,*]

[1]Division of Biomedical Sciences, School of Medicine, University of California, Riverside, Riverside, CA 92521, USA
[2]Department of Quantitative and Computational Biology, University of Southern California, Los Angeles, CA 90089, USA

*Correspondence to Sika Zheng: Division of Biomedical Sciences, School of Medicine, University of California, Riverside, Riverside, CA, 92521. sika.zheng@ucr.edu
T: 1-951-827-7670; F: 1-951-579-4118

Supplementary Figure 1. Expression of axonal markers and TRIM46 in ESC-derived neurons.
Supplementary Figure 2. mRNA expression of *Trim46* and inclusion levels of exons 8 and 10 in embryonic mouse cortex.
Supplementary Figure 3. PTBP2 CLIP of *Trim46*.
Supplementary Figure 4. TRIM46 antibodies effectively detect both isoforms.
Supplementary Figure 5. Endogenous TRIM46S protein is not detected in supernatant or pellet fractions.
Supplementary Figure 6. TRIM46S protein isoform is not stable.
Supplementary Figure 7. MG132 toxicity of ESC-neurons precludes detection of TRIM46S protein.
Supplementary Figure 8. Characterization of WT and mutant E10-KO ESC-derived neurons.
Supplementary Figure 9. Exon 8 included-*Trim46* transcripts are regulated by nonsense-mediated mRNA decay.
Supplementary Figure 10. Characterization of WT and E8-KO mutant ESC-derived neurons.
Supplementary Figure 11. Exon 8 and Exon 10 Splicing Are Independently Regulated.
Supplementary Figure 12. PTBP2 does not regulate exon 8 splicing.

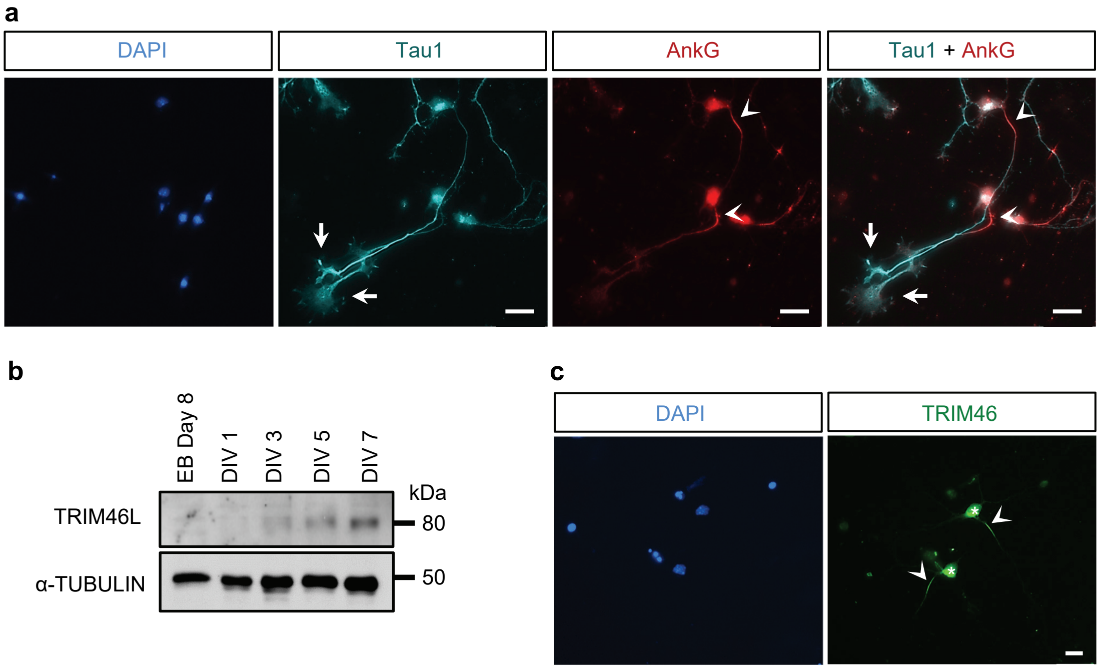

Supplementary Figure 1. Expression of axonal markers and TRIM46 in ESC-derived neurons. a. Immunostaining for Tau1 and AnkG in mature ESC-derived neurons on DIV 7 from 3 independent experiments. Arrowheads point to AIS staining and arrows indicate distal axon. Scale bars, 10 μm. **b.** TRIM46 protein expression during neuronal differentiation from EB stage to ESC-derived neurons. N = 2 shows the same results. **c.** Immunostaining for TRIM46 shows localization at the proximal axon from ESC-derived neurons on DIV 3 from 6 independent experiments. Asterisk indicates nonspecific TRIM46 somatic staining and arrowheads point to axon specific staining. Scale bar, 10 μm.

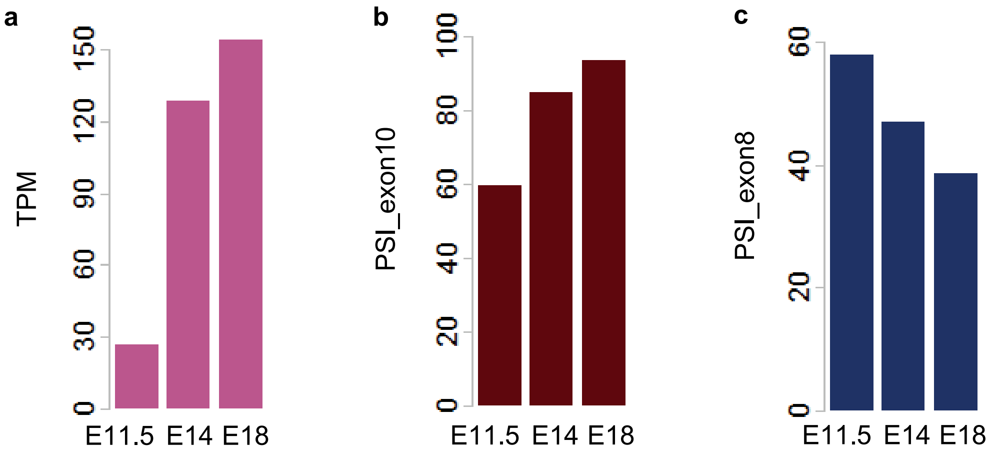

Supplementary Figure 2. mRNA expression of *Trim46* and inclusion levels of exons 8 and 10 in embryonic mouse cortex. a. Expression of total *Trim46* mRNA expression (TPM) in mouse cortices at ages E11.5 (SRX186052), E14 (SRX186048), and E18 (SRX186049). RNA seq data was obtained from ENCODE (Thomas Gingera's lab). The same dataset was used to calculate **b**. exon 10 inclusion (%) and **c**. exon 8 inclusion (%).
.

a

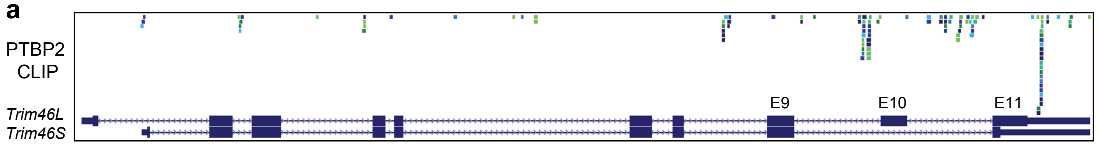

Supplementary Figure 3. PTBP2 CLIP of *Trim46.* a. Genome browser track for E18.5 PTBP2 CLIP-Seq of *Trim46* gene (mm9). Exons 9, 10 (alternative), and 11 are annotated along with two isoforms *Trim46L* (exon 10 included) and *Trim46S* (exon 10 skipped).

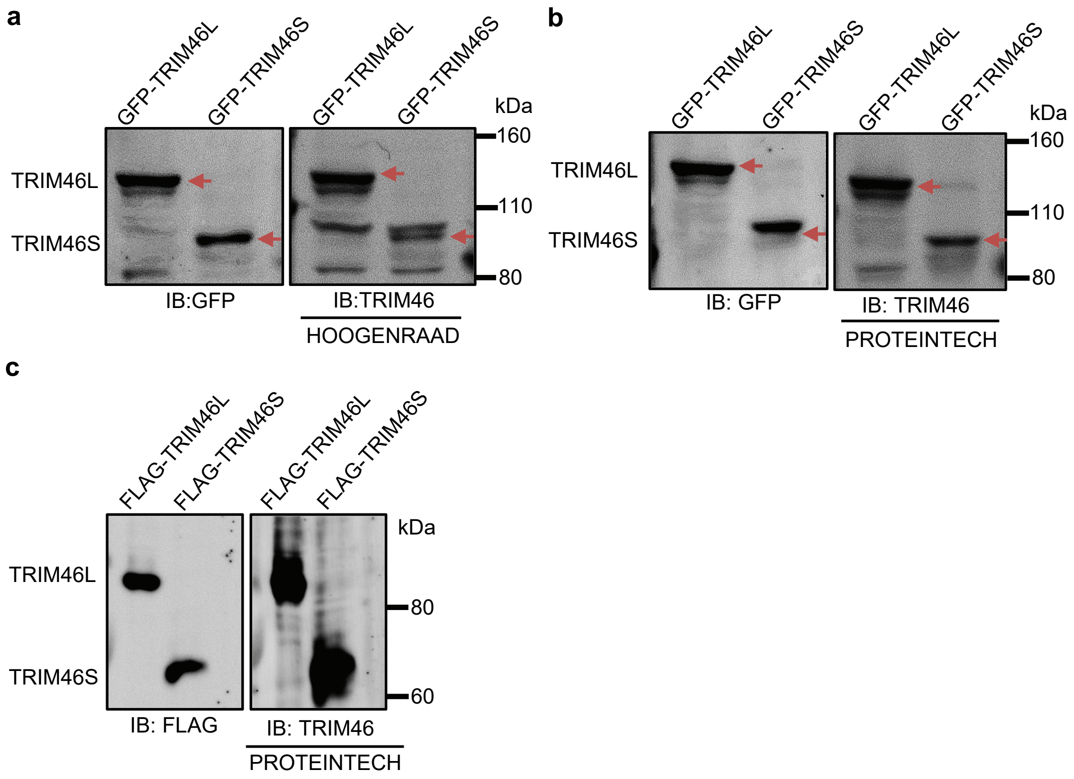

Supplementary Figure 4. TRIM46 antibodies effectively detect both isoforms. a., **b.** N2a cells expressing GFP-TRIM46 proteins were used to evaluate TRIM46-specific antibodies. Immunoblot for TRIM46 using antibodies from Hoogenraad lab and Proteintech. GFP immunoblots of the same gel were used as references. Red arrows point to GFP-TRIM46L and GFP-TRIM46S proteins of 120 kDa and 97 kDa, respectively. N = 2 independent experiments show same results. **c.** Western blot using FLAG and Proteintech TRIM46 antibodies to test FLAG-TRIM46 protein isoforms expressing in N2a cells. Expected protein sizes of FLAG-TRIM46L and FLAG-TRIM46S proteins are 84 kDa and 61 kDa, respectively. N = 4 shows same results.

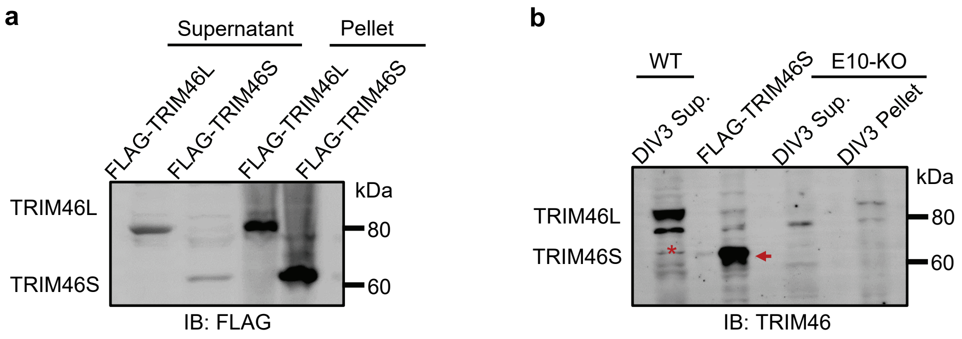

Supplementary Figure 5. Endogenous TRIM46S protein is not detected in supernatant or pellet fractions. **a**. Immunoblot for FLAG-TRIM46 proteins in supernatant and pellet. For supernatant fractions, 1.9% and 1.1% was loaded for FLAG-TRIM46L and FLAG-TRIM46S proteins, respectively. For pellet fractions, 57% and 61% was loaded for FLAG-TRIM46L and FLAG-TRIM46S proteins, respectively. N = 2 independent experiments with same results. The total % of TRIM46L protein present in supernatant fraction is 94% while the total % of TRIM46S protein is 89%. **b**. Western blot for TRIM46 proteins from DIV3 ESC-derived neurons; N2a cells expressing FLAG-TRIM46S (serving a postive control), as well as supernatant and pellet fractions of DIV 3 E10-KO neurons. Arrow points to FLAG-TRIM46S protein and asterisk (*) indicates a nonspecific band. N = 2 shows same results.

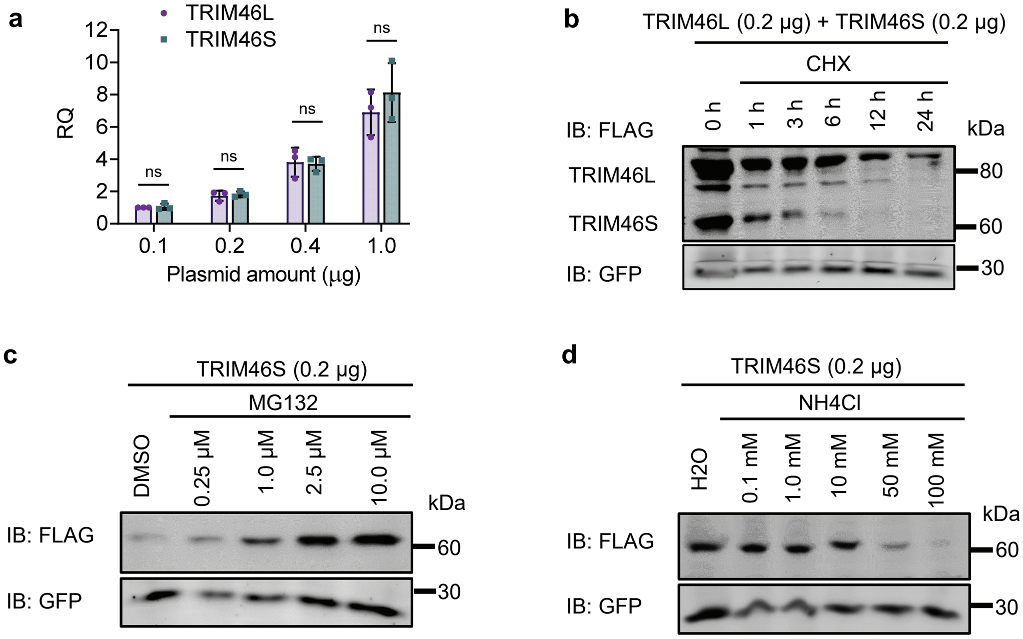

Supplementary Figure 6. TRIM46S protein isoform is not stable. **a**. RT-qPCR shows FLAG-*Trim46* isoforms are not differentially expressed under transfected dosages of 0.1 to 1.0 μg in N2a cells. Data is represented as mean ± STD: TRIM46L v TRIM46S; 0.1 μg, ns = 0.705063101, 0.2 μg, ns = 0.67389884, 0.4 μg ns = 0.864529574, 1.0 μg, ns = 0.409375818; n = 3 biological replicates. **b**. Co-expressed in N2a cells, FLAG-TRIM46S protein drastically decreased over time in comparison to FLAG-TRIM46L protein under translation inhbiiton (CHX treatment) from 2 independent experiments. **c**. FLAG-TRIM46S proteins expressed in N2a cells are upregulated with increasing MG132 dosage. N = 2 shows similar results. **d**. FLAG-TRIM46S proteins expressed in N2a cells are largely unaffected by lower concentrations of NH4Cl but substantially decreased in higher dosages from 2 independent experiments.

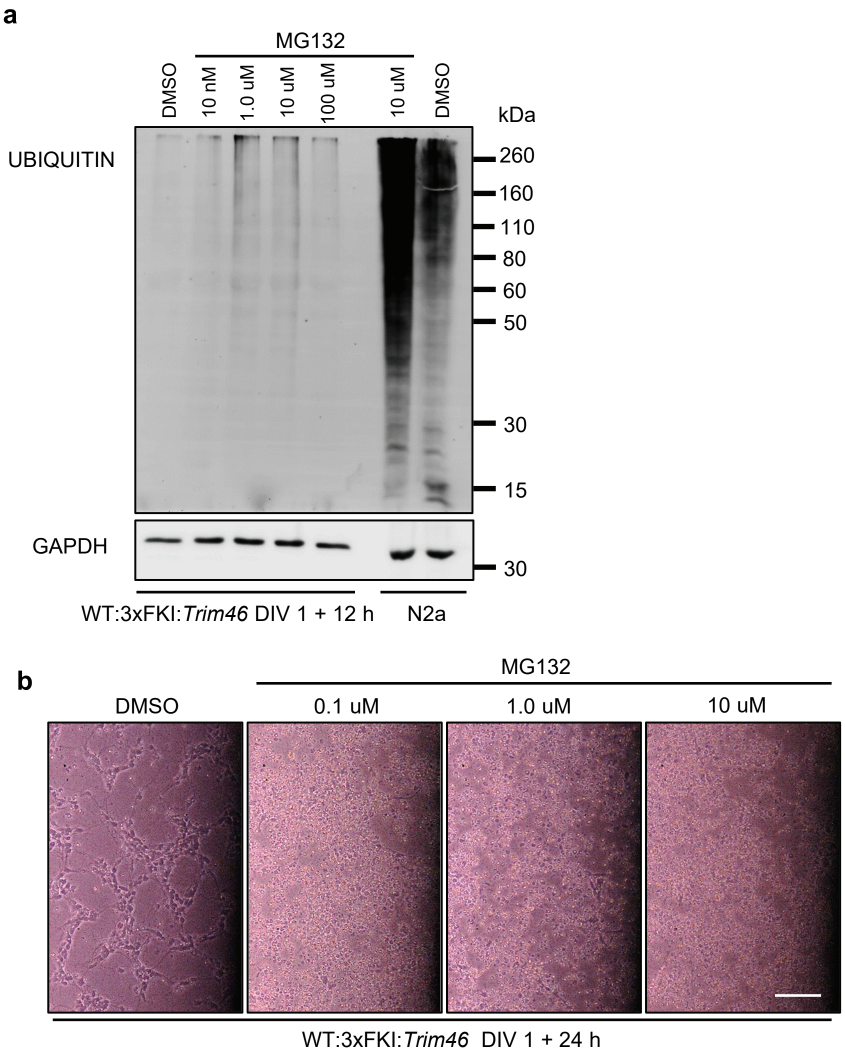

Supplementary Figure 7. MG132 toxicity of ESC-neurons precludes detection of TRIM46S protein.
a. Immunoblot for UBIQUITIN shows modest proteasome inhibition in MG132 treated 3xFLAG-KI-neurons and strong proteasome inhibition in N2a cells (positive control); N = 2 with similar results. **b**. Images of 3xFLAG-KI-neurons on DIV 1 post-treatment with various concentrations of MG132 for 24 hours; N = 2 independent experiments with same results. Scale bar, 50 um.

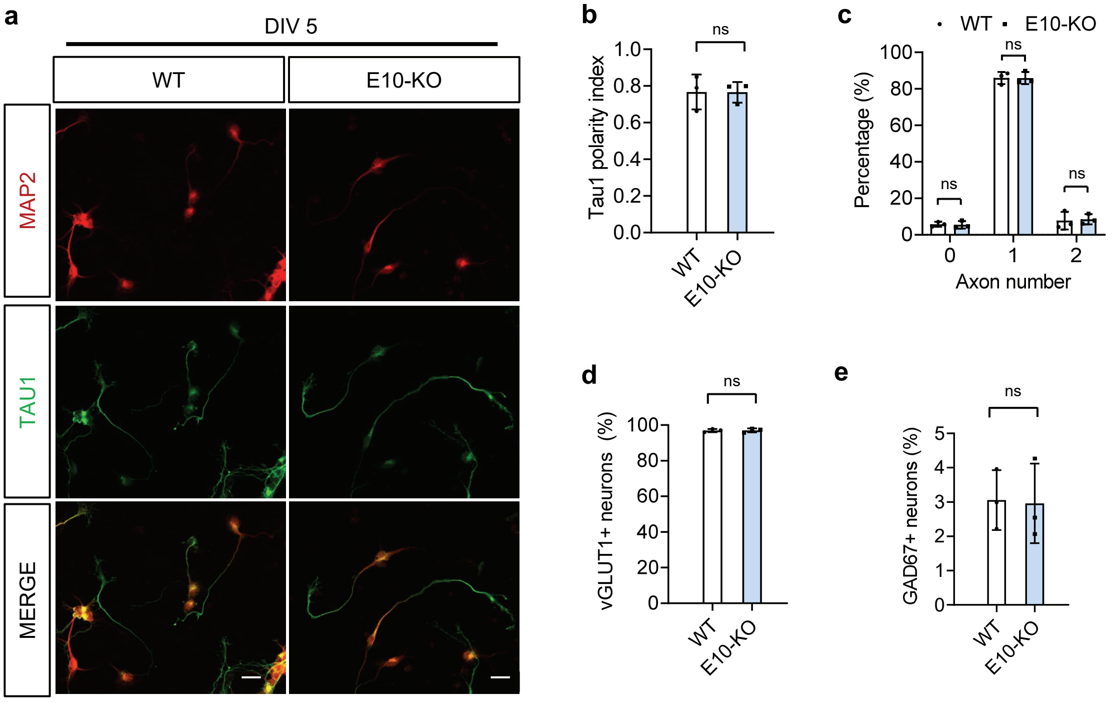

Supplementary Figure 8. Characterization of WT and mutant E10-KO ESC-derived neurons.
a. Immunostaining for MAP2 and Tau1 from ESC-derived neurons on DIV 5. Scale bars, 20 μm. **b., c.** Tau1 polarity index and quantification of axon number for WT and E10-KO neurons on DIV 5. Data is represented as mean ± SD; Statistics: t-test, two-tailed, unpaired; Tau1 index; WT v. E10-KO, ns = 0.954313976. Axon number; WT v. E10-KO; axon 0, ns = 0.8724546, axon 1, ns = 0.867485644, axon 2 = 0.827660689; WT, n = 167 and E10-KO, n = 130 from 3 independent experiments. **d**., **e**. Quantification for vGLUT1 and GAD67-positive staining between WT and E10-KO neurons on DIV 12. Data is represented as mean ± SD; Statistics: t-test, two-tailed, unpaired: vGLUT1 and GAD67, WT v. E10-KO, * = 0.912653802; WT, n = 638 and E10-KO, n = 542 from 3 independent experiments.

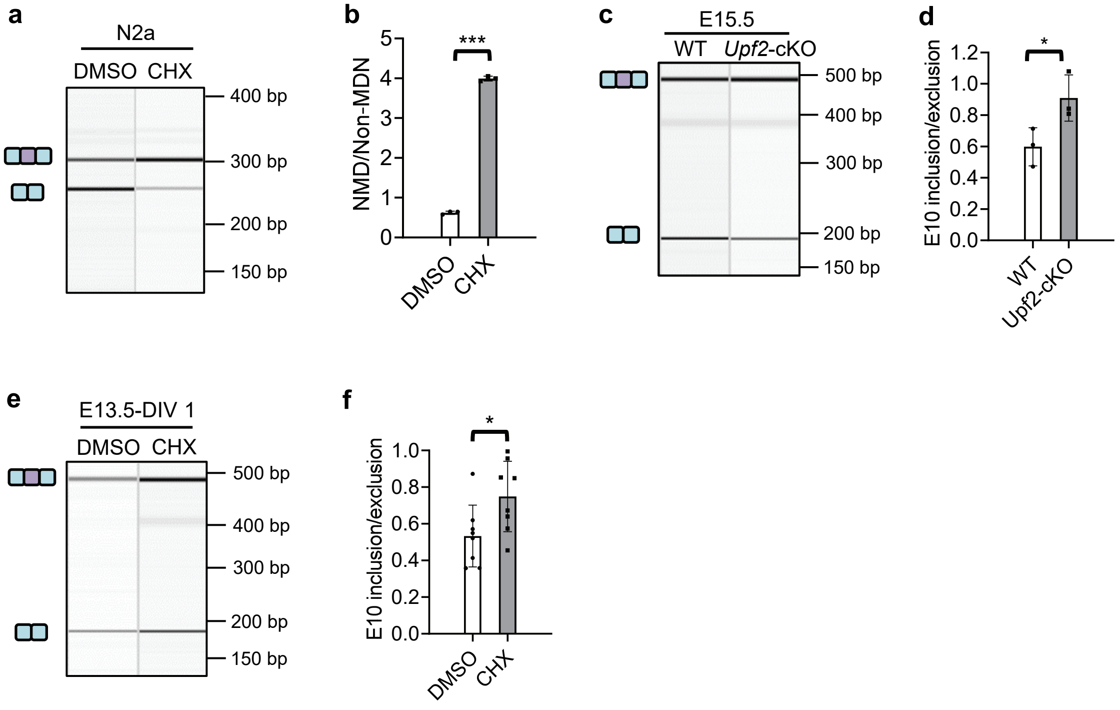

Supplementary Figure 9. Exon 8 included-*Trim46* transcripts are regulated by nonsense-mediated mRNA decay.
a. Gel image shows *Trim46* E8+ isoform increases significantly in N2a cells under CHX treatment or NMD repression. **b.** Graph highlights substantial increase of NMD-sensitive transcripts. Data is represented as mean ± STD; Statistics: t-test, two-tailed, unpaired; DMSO and CHX, n = 3 independent experiments; ***p = 1.39749x10^-7. **c.**, **d**. E15.5 cortex exhibits slight increase in E10+ isoform under depletion of *Upf2*. Data is represented as mean ± STD; Statistics: t-test, two-tailed, unpaired; E15.5 WT and *Upf2*-cKO, n = 3; *p = 0.047743787. **e.**, **f**. E13.5-DIV 1 primary cortical neurons treated with CHX shows mild increase of E10+ isoform. Data is represented as mean ± STD; Statistics: t-test, two-tailed, unpaired; E13.5-DIV1 of DMSO and CHX, n = 8; *p = 0.031617082.

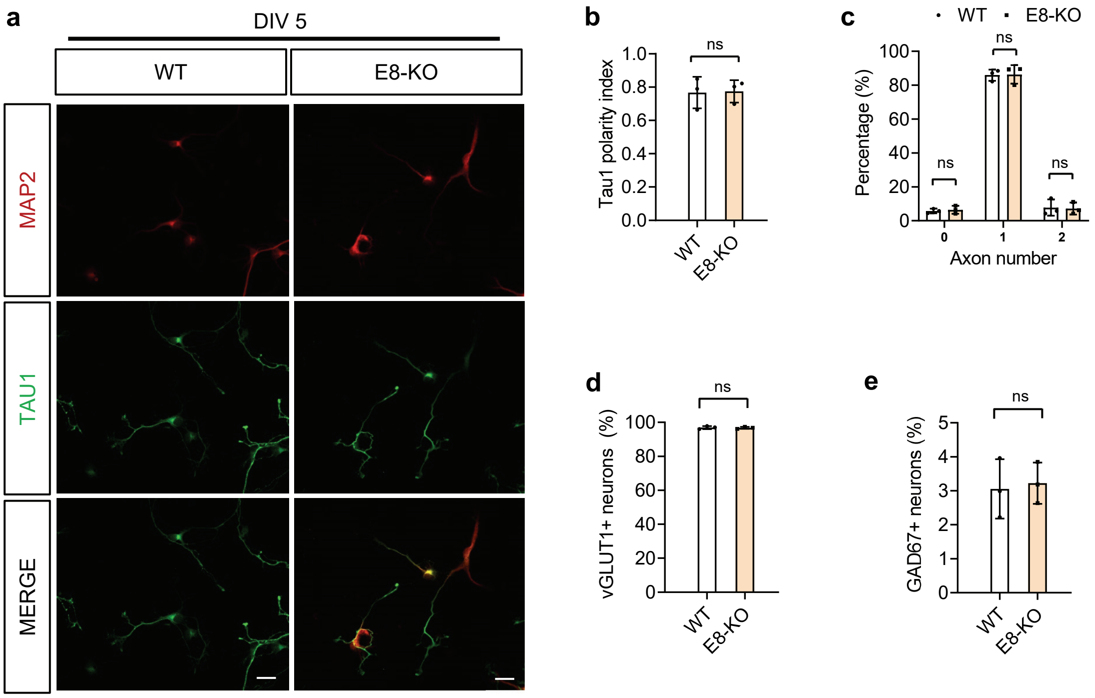

Supplementary Figure 10. Characterization of WT and E8-KO mutant ESC-neurons.

a. Immunostaining for MAP2 and Tau1 from ESC-derived neurons on DIV 5. Scale bars, 20 µm. **b**. Tau1 polarity index in WT and E8-KO ESC-derived neurons at DIV 5. Data is represented as mean ± SD; Statistics: t-test, two-tailed, unpaired; WT v. E8-KO, ns = 0.809655715; N = 3 independent experiments with similar results. **c.** Quantification of axon number for WT and mutant ESC-neurons lacking exon 8. Data is represented as mean ± SD; Statistics: t-test, two-tailed, unpaired: WT v. E8-KO; axon 0, ns =0.681462017 , axon 1, ns =0.979553592 , axon 2 =0.872863524; WT, n = 167 and E8-KO, n =140 from 3 biological replicates. **d., e.** Quantification for vGLUT1 and GAD67-positive staining between WT and E8-KO neurons on DIV 12. Data is represented as mean ± SD; Statistics: t-test, two-tailed, unpaired: vGLUT1 and GAD67, WT v. E8-KO, ns = 0.799115372; WT, n = 638 and E8-KO, n = 595 from 3 biological replicates with same results.

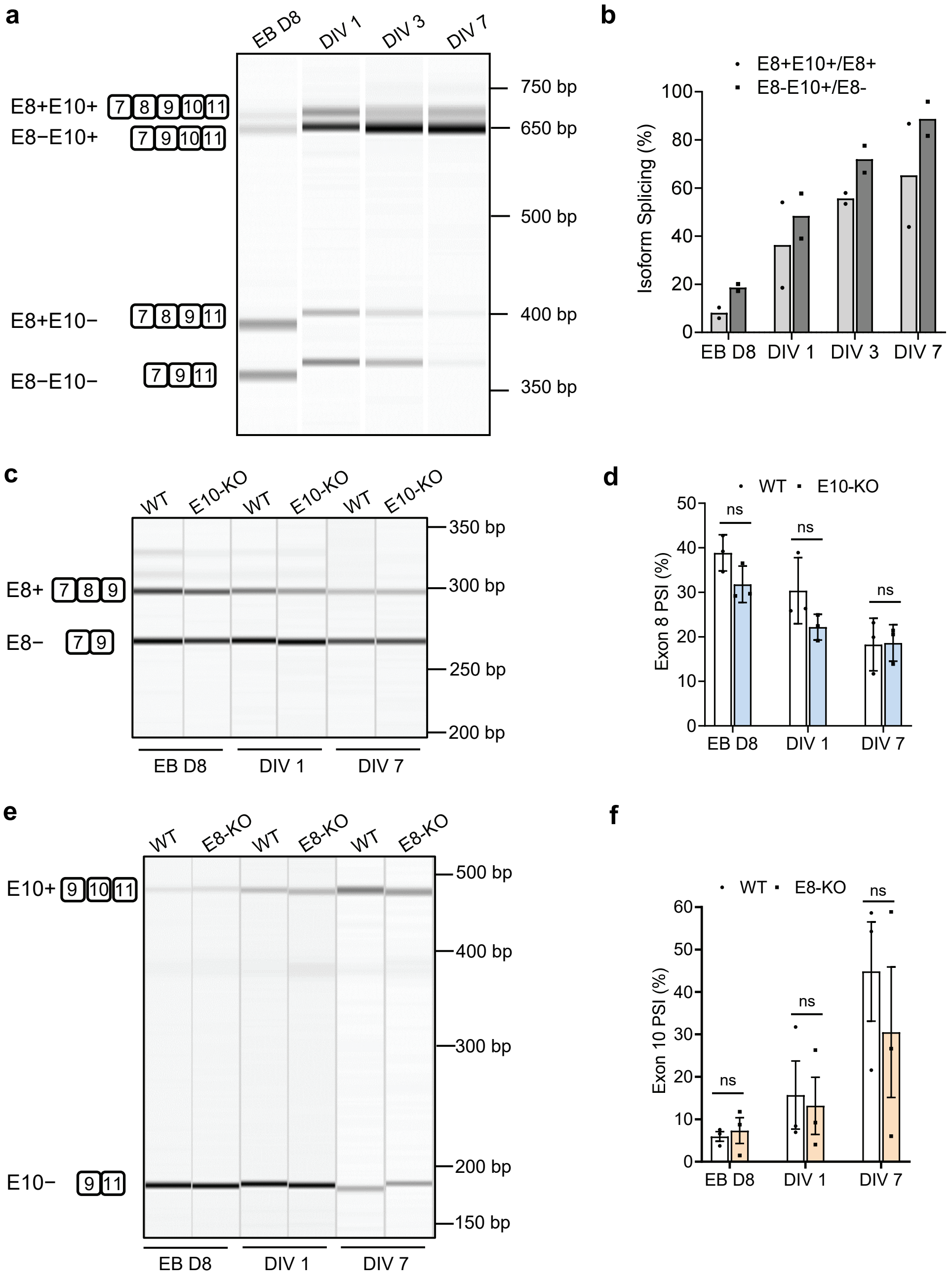

Supplementary Figure 11. Exon 8 and Exon 10 Splicing are Independently Regulated.

a. Alternative Trim46 isoforms containing and skipping exons 8 and 10 in WT ESC-derived neurons during axon formation. **b**. Graph demonstrates exon 10 inclusion level in exon 8-containing, and exon 8-skipping mRNA transcripts from EB day 8 to DIV 7. Data are from n = 2 independent experiments with same results. **c**. Exon 8 splicing in WT and mutant E10-KO neurons over multiple stages. **d**. Removal of exon 10 does not lead to differences in E8 splicing from EB day 8 to DIV 7. Data is represented as mean ± STD; Statistics: t-test, two-tailed, unpaired; WT v E10-KO; EB D8, ns = 0.10117961, DIV 1, ns = 0.148777386, and DIV 7, ns = 0.935454024; WT and E10-KO, n = 3 independent experiments with similar results. **e**. Exon 10 splicing in WT and mutant E8-KO neurons during neuronal differentiation. **f**. Deletion of exon 8 does not lead to differences in E10 splicing between control and exon 8 KO mutants across multiple tested stages. Data is represented as mean ± SEM; Statistics: t-test, two-tailed, unpaired; WT v E8-KO, EB D8, ns = 0.692719709, DIV 1, ns = 0.821371839, and DIV 7, ns = 0.49964825; WT and E8-KO, n = 3 independent experiments with similar results.

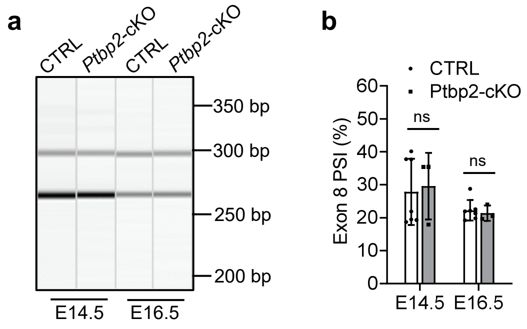

Supplementary Figure 12. PTBP2 does not regulate exon 8 splicing.
a. Gel image of NMD and non-NMD isoforms from CTRL and *Ptbp2*-cKO cortex on E14.5 and E16.5; n = 3 independent experiments with same results. **b.** Graph shows no change in exon 8 splicing in PTBP2 conditional knockout cortices. Data is represented as mean ± STD: Statistics: t-test, two-tailed, unpaired; CTRL v *Ptbp2*-cKO; E14.5, ns = 0.807616753, CTRL n = 7, *Ptbp2*-cKO n = 3; E16.5, ns = 0.677386575; E16.5 CTRL, n = 7, *Ptbp2*-cKO, n = 3.
