# Supplementary material for: Multilayered regulations of alternative splicing, NMD, and protein stability control temporal induction and tissue-specific expression of TRIM46 during axon formation
Source: Nat Commun. 2022 Apr 19;13:2081. doi: 10.1038/s41467-022-29786-4 (PMC9019110; doi:10.1038/s41467-022-29786-4)
Supplement: Supplementary file 3 — Description of Additional Supplementary Files [file 41467_2022_29786_MOESM3_ESM.pdf]

Description of Additional Supplementary Files

File Name: Supplementary Data 1
Description: List of primers
