# Supplementary material for: Multilayered regulations of alternative splicing, NMD, and protein stability control temporal induction and tissue-specific expression of TRIM46 during axon formation
Source: Nat Commun. 2022 Apr 19;13:2081. doi: 10.1038/s41467-022-29786-4 (PMC9019110; doi:10.1038/s41467-022-29786-4)
Supplement: Supplementary file 5 — Reporting Summary [file 41467_2022_29786_MOESM5_ESM.pdf]

nature research

Corresponding author(s): Sika Zheng

Last updated by author(s): 02/15/2022

Reporting Summary

Nature Research wishes to improve the reproducibility of the work that we publish. This form provides structure for consistency and transparency in reporting. For further information on Nature Research policies, see our Editorial Policies and the Editorial Policy Checklist.

Statistics

For all statistical analyses, confirm that the following items are present in the figure legend, table legend, main text, or Methods section.

n/a	Confirmed	
☐	☒	The exact sample size (*n*) for each experimental group/condition, given as a discrete number and unit of measurement
☐	☒	A statement on whether measurements were taken from distinct samples or whether the same sample was measured repeatedly
☐	☒	The statistical test(s) used AND whether they are one- or two-sided<br>*Only common tests should be described solely by name; describe more complex techniques in the Methods section.*
☒	☐	A description of all covariates tested
☒	☐	A description of any assumptions or corrections, such as tests of normality and adjustment for multiple comparisons
☐	☒	A full description of the statistical parameters including central tendency (e.g. means) or other basic estimates (e.g. regression coefficient) AND variation (e.g. standard deviation) or associated estimates of uncertainty (e.g. confidence intervals)
☐	☒	For null hypothesis testing, the test statistic (e.g. *F*, *t*, *r*) with confidence intervals, effect sizes, degrees of freedom and *P* value noted<br>*Give P values as exact values whenever suitable.*
☒	☐	For Bayesian analysis, information on the choice of priors and Markov chain Monte Carlo settings
☒	☐	For hierarchical and complex designs, identification of the appropriate level for tests and full reporting of outcomes
☒	☐	Estimates of effect sizes (e.g. Cohen's *d*, Pearson's *r*), indicating how they were calculated

Our web collection on statistics for biologists contains articles on many of the points above.

Software and code

Policy information about availability of computer code

Data collection	Nikon BR4.5 software was used quantify AnkG, TRIM46, Tau1, vGLUT1, and GAD67 immunostaining data (Fig. 4f-i., Fig. 6i-n, Supplementary Fig. 8a-e and Supplementary Fig. 10a-e).<br>QIAxcel SceenGel 1.6.0 software was used to quantify RT-PCR results (Fig. 1g-h, Fig. 5c-h, Supplementary Fig. 9a-f, Supplementary Fig. 11a-f and Supplementary Fig. 12a-b).<br>ImageQuantTL-8.1 software was used to quantify western blot results (Fig. 1c-d, Fig. 3a-f, and Fig. 6d-g).<br>QuantStudio Real-Time PCR Software v1.7.1 (Fig. 4e, Fig. 6h, and Supplementary Fig. 6a).
Data analysis	All analysis was performed using Microsoft Excel 2016 Version 2201 by Student's t-test under one-tailed or two-tailed, unpaired or paired conditions and evaluated by various p-values while half-life was calculated by GraphPad Prism version 8.02 (263).

For manuscripts utilizing custom algorithms or software that are central to the research but not yet described in published literature, software must be made available to editors and reviewers. We strongly encourage code deposition in a community repository (e.g. GitHub). See the Nature Research guidelines for submitting code & software for further information.

Data

Policy information about availability of data

All manuscripts must include a data availability statement. This statement should provide the following information, where applicable:

- Accession codes, unique identifiers, or web links for publicly available datasets
- A list of figures that have associated raw data
- A description of any restrictions on data availability

The data that support findings in this study are available upon request.

Field-specific reporting

Please select the one below that is the best fit for your research. If you are not sure, read the appropriate sections before making your selection.

☒ Life sciences ☐ Behavioural & social sciences ☐ Ecological, evolutionary & environmental sciences

For a reference copy of the document with all sections, see nature.com/documents/nr-reporting-summary-flat.pdf

Life sciences study design

All studies must disclose on these points even when the disclosure is negative.

Sample size	Biological replicates represent individual (n) sample size for experiments involving mouse cortices, primary cortical neurons, ESC differentiation, and N2a cells. For immunoblotting experiments of ESC-derived neurons, biological replicates across different dates represent individual (n) for sample size while for cell staining experiments sample size (n) was determined for individual cells quantified in coverslips and biological replicates are also declared.
Data exclusions	No data was excluded from analysis.
Replication	All experiments were performed minimum as biological duplicates as independent experiments occurring at different dates and consistent.<br>Biological replicates of Ptbp2-cKO/Upf2-cKO neocortices were minimum 2 and maximum 7.<br>For primary cortical neurons, RT-PCR analysis included 5 and 8 biological replicates.<br>For ESC-derived neurons western blot data included 4 biological replicates.<br>For E8 and E10-KO cell staining analysis for ESC-neurons included triplicates but also included total between 130-638 individual cells.<br>N2a experiments were performed mostly in triplicates and minimum duplicates with all consistent results. All experiments were well defined and highly standardized to produce consistent results.
Randomization	Data collected for analysis was not randomized.
Blinding	Blinding was not performed in this study.

Reporting for specific materials, systems and methods

We require information from authors about some types of materials, experimental systems and methods used in many studies. Here, indicate whether each material, system or method listed is relevant to your study. If you are not sure if a list item applies to your research, read the appropriate section before selecting a response.

Materials & experimental systems

n/a	Involved in the study	
☐	☒	Antibodies
☐	☒	Eukaryotic cell lines
☒	☐	Palaeontology and archaeology
☐	☒	Animals and other organisms
☒	☐	Human research participants
☒	☐	Clinical data
☒	☐	Dual use research of concern

Methods

n/a	Involved in the study	
☒	☐	ChIP-seq
☒	☐	Flow cytometry
☒	☐	MRI-based neuroimaging

Antibodies

Antibodies used	TRIM46: Gift from Hoogenraad lab; Proteintech Inc. (21026-1-AP)<br>Tau1: Millipore (MAB3420), clone PC1C6<br>MAP2: Abcam (ab5392)<br>AnkG: neuroMab (75-146); Synaptic Systems (386005), clone N106/36<br>vGLUT1: Synaptic Systems (135302)<br>GAD67: Millipore (MAB5406), clone 1G10.2<br>FLAG: Sigma-Aldrich (F7425)<br>UBIQUITIN: (Cell Signaling, 3933)<br>GFP: Aves (GFP-1020)<br>GAPDH: ThermoFisher (AM4300)<br>α-TUBULIN: Calbiochem (CP06-100µg), clone DM1A<br>IgG control: Proteintech Inc. (30000-0-AP)
Validation	TRIM46-Hoogenraad (rb): (van Beuningen et. al; WB & IF/ICC, 2015 Neuron)<br>TRIM46-Proteintech Inc. (rb): https://www.ptglab.com/products/TRIM46-Antibody-21026-1-AP.htm<br>Tau1-Millipore (m): https://www.emdmillipore.com/US/en/product/Anti-Tau-1-Antibody-clone-PC1C6,MM_NF-MAB3420

MAP2-Abcam (chk): https://www.abcam.com/map2-antibody-ab5392.html
AnkG-neuroMab (m): https://neuromab.ucdavis.edu/datasheet/N106_36.pdf
AnkG-Synaptic Systems (gp): https://sysy.com/product/386005
vGLUT1-Synaptic Systems (rb): https://sysy.com/product/135302
GAD67-Millipore (m): https://www.emdmillipore.com/US/en/product/Anti-GAD67-Antibody-clone-1G10.2,MM_NF-MAB5406
FLAG-Sigma Aldrich (rb): https://www.sigmaaldrich.com/catalog/product/sigma/f7425?lang=en&region=US
UBIQUITIN-Cell Signaling (rb): https://www.cellsignal.com/products/primary-antibodies/ubiquitin-antibody/3933
GFP-Aves (chk): https://www.aveslabs.com/products/green-fluorescent-protein-gfp-antibody?variant=25144111202404
GAPDH (m): https://www.thermofisher.com/antibody/product/GAPDH-Antibody-clone-6C5-Monoclonal/AM4300
α-TUBULIN-Calbiochem (m): https://www.emdmillipore.com/US/en/product/Anti-Tubulin-Mouse-mAb-DM1A,EMD_BIO-CP06
IgG control (rb): https://www.ptglab.com/products/IgG-control-Antibody-30000-0-AP.htm

Eukaryotic cell lines

Policy information about cell lines

Cell line source(s)	N2a, 46C mESC
Authentication	N2a acquired from ATCC (#CCL-131) and 46C mES cells acquired from USC group (Qi-Long Ying) and authenticated by Sox1-GFP expression.
Mycoplasma contamination	Cell lines were not tested for Mycoplasma contamination.
Commonly misidentified lines (See ICLAC register)	No misidentified lines were used in this study.

Animals and other organisms

Policy information about studies involving animals; ARRIVE guidelines recommended for reporting animal research

Laboratory animals	Embryonic and post-natal mice of control and Ptbp2-cKO or Upf2-cKO are described in methods section.
Wild animals	No wild animals were used in this study
Field-collected samples	This study did not collect field samples.
Ethics oversight	Guidelines of Institutional Animal Care and Use Committees (IACUC) of UC Riverside were approved and followed as described in the methods section.

Note that full information on the approval of the study protocol must also be provided in the manuscript.
